# TerminatorNet: comprehensive identification of intrinsic transcription terminators in bacteria

**DOI:** 10.1093/bioinformatics/btag116

**Published:** 2026-03-11

**Authors:** Brian Tjaden

**Affiliations:** Department of Computer Science, Wellesley College, Wellesley, MA 02481, United States

## Abstract

**Motivation:**

The primary mechanism for transcription termination in bacteria is intrinsic terminators. These terminators influence transcript stability and play key roles in gene regulation. Existing computational methods for genome-wide terminator identification have been designed and evaluated based on a small number of experimentally evinced terminators often from only one or two organisms.

**Results:**

We present TerminatorNet, a system for identifying intrinsic transcription terminators throughout bacteria. TerminatorNet uses a neural network model trained on a large set of experimentally characterized transcription terminators from a variety of bacterial genomes. TerminatorNet identifies 98% of terminators and has a false positive rate of 3%, substantially better than existing approaches. TerminatorNet commonly identifies terminators at the ends of operons. We applied TerminatorNet to thousands of genomes across the taxonomic spectrum of prokaryotes, creating a repository of tens of millions of terminators. We observe heavy use of intrinsic termination in some groups, such as Bacillota, and rare use in other groups such as archaea. We also observe a wealth of instances of DNA uptake signal sequences, important components of transformation specificity for some competent bacteria, in terminators identified in *Neisseriaceae* and *Pasteurellaceae*.

**Availability and implementation:**

TerminatorNet and its repository of identifications are available for use via a webserver: https://cs.wellesley.edu/~btjaden/TermNet. The source code is available at GitHub https://github.com/btjaden/TerminatorNet and Zenodo https://doi.org/10.5281/zenodo.18406126.

## Graphical abstract

**Figure:**
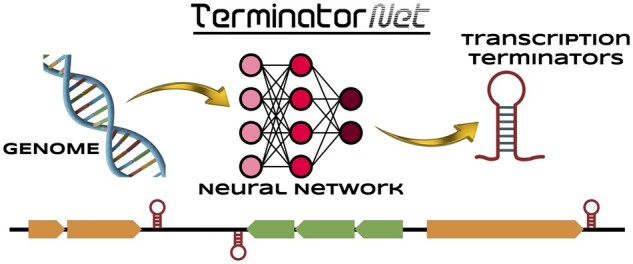

## 1 Introduction

Transcription termination in bacteria is the process by which RNA polymerase halts RNA synthesis and dissociates from the DNA template, releasing the newly formed RNA transcript. Termination not only is essential for ending transcription at the correct position, but also plays a key role in transcript stability and in gene regulation. Improper termination can lead to readthrough transcription, antisense RNA production, and interference with downstream gene expression (Peters *et al.* 2011). Furthermore, bacteria often exploit differences in termination efficiency as part of regulatory strategies (Ray-Soni *et al.* 2016). A classic example of the involvement of transcription termination in regulation is attenuation of the *trp* operon in *Escherichia coli* where, when tryptophan is abundant, the ribosome is less likely to stall and a hairpin structure forms in the leader region of the operon causing premature transcription termination (Bertrand *et al.* 1976). Identifying transcription terminators is an important component in annotating genes, characterizing operon structures, determining untranslated regions (UTRs), and understanding gene expression and regulation.

Bacterial transcription termination primarily occurs through two mechanisms: intrinsic (Rho-independent or factor-independent) termination and Rho-dependent termination. Intrinsic termination normally involves a stable hairpin loop structure followed by a short (tail) sequence rich in uracil residues. The formation of the hairpin in the RNA causes a physical disruption in the elongation complex (Gusarov and Nudler 1999). The weak base pairings between the RNA uracils and the DNA template adenines is less stable than other base pairings. The combination of mechanical stress from the hairpin and weak base pairing facilitates the dissociation of RNA polymerase from the DNA, terminating transcription (Santangelo and Artsimovitch 2011). Further, the hairpin structure helps protect the RNA from degradation. Intrinsic termination is efficient and widely used by bacteria.

As a complement to intrinsic termination, Rho-dependent termination requires the action of a specialized protein called Rho, a hexameric ATP-dependent RNA helicase (Song *et al.* 2022). Rho binds to the nascent RNA at a Rho utilization (rut) site, a C-rich, G-poor region that lacks secondary structure. Once bound, Rho translocates along the RNA in the 5′ to 3′ direction, using energy from ATP hydrolysis. Rho catches up to the RNA polymerase when the latter pauses at specific termination sites. Upon contact, Rho uses its helicase activity to unwind the RNA–DNA hybrid in the transcription bubble, leading to release of the RNA and disassembly of the transcription complex (Said *et al.* 2021). In addition to this traditional model of Rho-dependent termination, an alternative model has been observed where Rho first forms a pre-termination complex with RNA polymerase and elongation factors NusA and NusG prior to interacting with RNA as part of the termination process (Hao *et al.* 2021).

Intrinsic termination appears to be significantly more common among bacteria than Rho-dependent termination (Ciampi 2006). While Rho is generally conserved, it plays a lesser role in some organisms such as *Bacillus subtilis* (Bidnenko *et al.* 2017), whereas it is essential in other organisms such as *Salmonella enterica* (Langridge *et al.* 2009). Historically, intrinsic termination has not been described as utilizing factors such as Rho, however recent work has demonstrated that some intrinsic terminators indeed rely on factors such as Rho and NusA and NusG for efficient termination (Mandell *et al.* 2022) and, thus, our motivation for using the term “intrinsic” terminator rather than “Rho-independent” terminator. In the current study, we focus on intrinsic termination, as it is the most prevalent mechanism of transcription termination (Roberts 2019).

Transcriptional terminators are increasingly being characterized in bacteria thanks, in part, to high-throughput experimental approaches such as Term-seq. Term-seq is a next-generation RNA sequencing technique used to map the 3′ ends of RNA transcripts genome-wide, thereby identifying transcription termination sites with high precision (Dar *et al.* 2016). Term-seq has revealed widespread transcription termination across bacterial genomes, not only for protein coding genes and polycistronic operons, but also for antisense RNAs and small regulatory RNAs (sRNAs) (D’Halluin *et al.* 2023, Bar *et al.* 2023). These high-throughput methods have enabled characterization of thousands of transcription terminators. However, experimental methods do not necessarily scale with the rapid pace of bacterial genome sequencing and annotation. For instance, recent studies that aggregated Term-seq data, identified Term-seq datasets from 13 different species (Kosinski *et al.* 2025) and from 22 different species (Ghahfarokhi *et al.* 2025). In contrast, there are millions of sequenced and annotated bacterial genomes in NCBI’s RefSeq database (Haft *et al.* 2018). As a result, a number of computational methods, which are generally more efficient than experimental methods, have been developed for rapid identification of transcription terminators in bacterial genomes.

For computational methods that predict intrinsic terminators based on genomic sequence information, rather than on high-throughput expression data, the features most commonly used to identify terminators are the free energy of the hairpin structure and some measure of the strength of the uracil-rich tail sequence following the hairpin structure (Lesnik *et al.* 2001, Naville *et al.* 2011, Millman *et al.* 2017, Brandenburg *et al.* 2022). Indeed, these are the two features used by perhaps the most popularly used computational method, TransTermHP (Kingsford *et al.* 2007). However, others have observed that there is only a weak correlation between the free energy of the hairpin and terminator efficiency, and instead have used other properties of the hairpin, including its leading adenine-rich head sequence (Chen *et al.* 2013), *k*-tuples (Feng *et al.* 2019), or a covariance model of the hairpin (Gardner *et al.* 2011) to predict terminators. Alternatively, rather than use a small number of features specifically related to terminators, one recent study explored whether over six thousand general genomic sequence features could help distinguish terminators from non-terminators (Ghahfarokhi *et al.* 2025).

In this study, we characterize 27 features indicative of intrinsic transcription terminators, and we train and evaluate a neural network model based on more than 10 000 terminators from a range of bacterial species. The large dataset of diverse terminators enables our deep learning model, TerminatorNet, to identify terminators with high accuracy, achieving 98% sensitivity, 97% specificity, and an AUC (area under ROC curve) of 0.99, significantly better than existing approaches. Given the efficiency of TerminatorNet, we use it to identify terminators in thousands of bacterial genomes, generating a repository of tens of millions of intrinsic transcription terminators, orders of magnitude larger than existing terminator databases as well as being more precise. The large repository of terminators enables us to identify species and other taxonomic groups that make lighter and heavier use of intrinsic terminators. Searching for motifs within our large database of terminators, we observe a number of DNA uptake signal sequences used as part of transformation in certain families of competent bacteria (Dubnau and Blokesch 2019, Arnold *et al.* 2022). In order to ensure reproducibility and facilitate use by the microbiology community, we have made our system available via a web interface, so that users can easily identify terminators in their own genomic sequences of interest as well as view and download identified terminators for any genome in the repository: https://cs.wellesley.edu/~btjaden/TermNet.

## 2 Methods

### 2.1 Datasets

For building and evaluating our system, we used data from an atlas of 11 769 experimentally validated intrinsic terminators aggregated from Term-seq datasets for 13 bacterial species (Kosinski *et al.* 2025). The data were split into training data used to build and tune the machine learning model and testing data used to evaluate the trained system. Training data include 6123 terminators from 11 bacterial species, *Listeria monocytogenes*, *Enterococcus faecalis*, *Streptomyces griseus*, *Streptomyces avermitilis*, *Streptomyces coelicolor*, *Streptomyces venezuelae*, *Streptomyces tsukubensis*, *Streptomyces lividans*, *Streptomyces clavuligerus*, *Zymomonas mobilis*, *Synechocystis* sp. PCC 6803, and testing data include 5646 terminators from 2 bacterial species, *B. subtilis* and *E. coli* (Table 1, available as supplementary data at *Bioinformatics* online). For corresponding sequences that do not contain terminators, we randomly sampled each terminator sequence according to its dinucleotide content so that its negative control sequence had the same expected dinucleotide content. Altogether, this resulted in 12 246 sequences for training data (Table 2, available as supplementary data at *Bioinformatics* online) and 11 292 sequences for testing data (Table 3, available as supplementary data at *Bioinformatics* online).

### 2.2 Machine learning analysis

Prior to training the machine learning model, data were normalized using feature scaling so that the set of values for each feature had a mean of zero and standard deviation of one. To assess the extent to which sequences corresponding to terminators could be distinguished from sequences not corresponding to terminators, a neural network model, specifically a multi-layer fully connected perceptron, was trained (Tjaden and Tjaden 2023). The neural network architecture consists of three trainable layers. The first two hidden layers have 50 units and 10 units, respectively, using a rectified linear unit (ReLU) activation function. The final output layer has 1 unit using a sigmoid activation function. Model parameters were determined by Adam stochastic optimization (Kingma 2014) for 1000 iterations with a batch size of 200 using the binary cross-entropy loss function. A constant learning rate was used with a step-size of 0.1. To help address possible overfitting, L2 regularization was used with a value of 0.0001. Altogether, the neural network model contained 1921 trainable parameters.

### 2.3 Performance evaluation

TerminatorNet’s performance in identifying experimentally validated intrinsic transcription terminators was compared to that of six other computational tools. RNIE was executed using its more accurate gene-based covariance model with command cmsearch -T 14 -g--fil-no-qdb --fil-no-hmm --no-qdb --inside gene.cm input.fa. termNN was executed with command python scan_genome.py using parameter values splits=50, ks=1, kernels_cutoff=0.5. TransTermHP was executed with command transterm -p expterm.dat input.fa input.coords. BacTermFinder was executed with command python genome_scan.py input.fa 3 10000. iTerm-PseKNC was executed with command python iTerm-PseKNC.py input.fa. ARNold was executed by uploading sequences to the ARNold webserver http://rssf.i2bc.paris-saclay.fr/toolbox/arnold. TerminatorNet was executed with command python TerminatorNet.py -f input.fa.

When considering transcription units and possible multi-gene operons, we identified a gene as a candidate for being the final gene of a (monocistronic or polycistronic) transcription unit if its downstream gene was on the opposite strand or if its downstream gene on the same strand was at least 1000 nucleotides downstream. We identified a gene as a candidate for being internal to a polycistronic transcription unit (not the final gene of a multi-gene operon) if its downstream gene on the same strand was at most 40 nucleotides downstream. A gene was considered to have a terminator if a terminator was identified within 100 nucleotides of the end of the gene, e.g. the stop codon of a protein coding gene. When searching for DNA uptake signal sequences, the frequency of every *k*-mer in a genome’s terminators was determined for *k *= 7, 8, 9, 10, 11, 12. The maximum frequency *k*-mer for each value of *k* was considered as a possible uptake signal sequence.

## 3 Results

### 3.1 Dataset of terminators

High-throughput experimental methods, such as Term-seq, enable the characterization of transcription terminators on a genome-wide scale. Here, we use data from an atlas of 11 769 experimentally validated intrinsic terminators aggregated from Term-seq datasets for 13 bacterial species (Kosinski *et al.* 2025). This large set of terminators provides a rich source of data for analysis by machine learning algorithms with the aims of identifying properties of the terminators and discerning terminators throughout genomes. As an initial step, we split the intrinsic terminators into two sets: a set of data consisting of 6123 terminators from 11 bacterial species for training our machine learning model and a set of data (out-of-sample) consisting of 5646 terminators from 2 bacterial species for evaluating our trained machine learning model (Table 1, available as supplementary data at *Bioinformatics* online). To avoid biases and data leakage, we deemed it important to separate the terminators based on species so that data used to evaluate our methods does not derive from any species used to build our machine learning model. Ultimately, this separation helps ensure that the results we report are likely to be representative of how the model performs on thousands of new species. The two species we included in our evaluative testing dataset are *B. subtilis* and *E. coli* because multiple Term-seq experiments have been conducted for each of these species and because they are not closely related to each other so we can assess, in part, how our method performs on different types of bacteria.

### 3.2 Features of terminators

**Figure 1:**
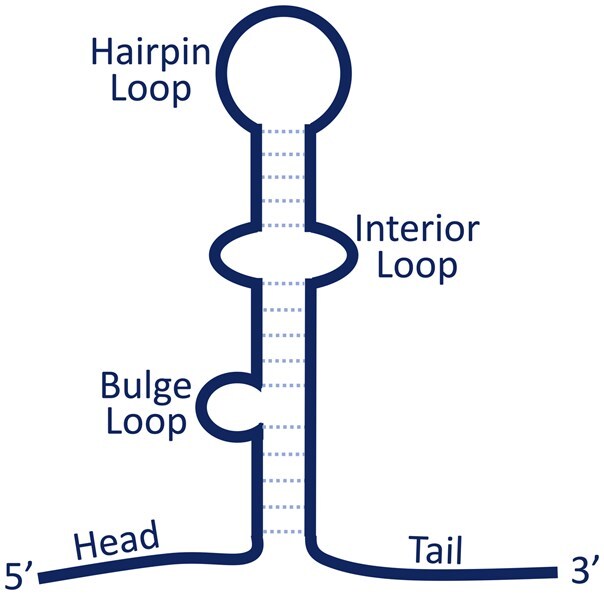
Illustrative components of an intrinsic terminator. The hairpin structure consists of a stem, possibly with bulge and interior loops, and a hairpin loop. Immediately upstream and downstream of the hairpin structure are the head and tail sequences, respectively.

Intrinsic terminators may have different components (Fig. 1). A variety of features have been used to identify these terminators computationally, with the most common features being the free energy of the hairpin structure and some measure of the uracil richness of the tail (Kingsford *et al.* 2007). As one example potential feature, 56% of validated terminators in our training dataset contain internal loops (interior loops or bulge loops) in their hairpin stem. We investigated 27 different features (Table 4, available as supplementary data at *Bioinformatics* online) and their prevalence in intrinsic terminators. In order to understand to what extent these features distinguish intrinsic terminators from similar genomic sequences that do not contain terminators, we generated negative (non-terminator) example sequences by randomly sampling each terminator sequence such that the corresponding negative example had the same expected dinucleotide content as the terminator sequence. Sampling disrupts the hairpin structure, while maintaining the dinucleotide content ensures the non-terminator sequence has reasonable statistical similarity to the corresponding terminator sequence and is a commonly used technique to generate negative controls for sequences relating to RNA structures (Workman and Krogh 1999). Thus, our training data consist of 12 246 sequences (Table 2, available as supplementary data at *Bioinformatics* online) and our testing data consists of 11 292 sequences (Table 3, available as supplementary data at *Bioinformatics* online), half of which are experimentally evinced intrinsic terminators and half of which are similar non-terminator sequences, and for each sequence we calculated values for each of the 27 features.

**Figure 2:**
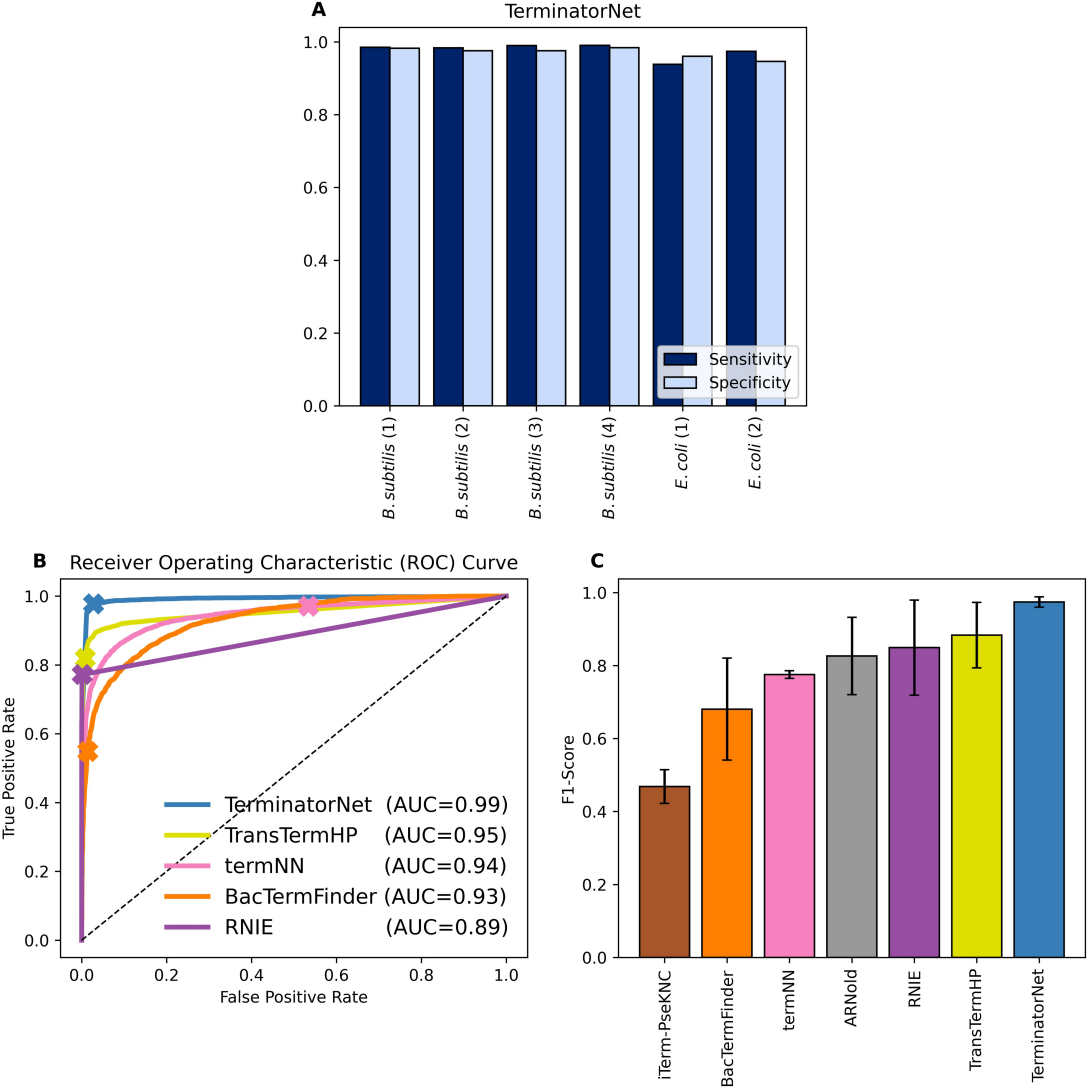
(A) The sensitivity and specificity of TerminatorNet is shown on the testing data. The testing data consist of terminators evinced from six different Term-seq experiments, four in *B. subtilis* and two in *E. coli*. (B) The ROC curves illustrate, for each of five computational tools, the trade-offs between true positive rate (sensitivity) and false positive rate (1.0 − specificity) for the tool on testing data, where each point along a curve corresponds to a different score threshold for the tool. The “X” along each curve indicates the true positive rate and false positive rate of the tool at its default score threshold value. The AUC for each tool is provided. (C) The average F1-score on testing data for seven computational tools is shown. The error bars reflect the standard deviation of the F1-score across the six Term-seq experiments that constitute the testing dataset.

To understand to what extent each feature differs among terminators and non-terminators, we visualized the distribution for each feature with respect to both terminators and non-terminators (Fig. 1, available as supplementary data at *Bioinformatics* online). For some features, such as the free energy of closure of the hairpin loop [Energy Loop (B)], the distributions for terminators and non-terminators appear similar, whereas for other features, such as the average free energy of the entire hairpin structure [Energy Hairpin Avg (B)], the distributions for terminators and non-terminators appear quite different. In order to quantify the dependency between each feature and whether a sequence corresponds to a terminator, we calculated the ANOVA (analysis of variance) F-statistic and coinciding *P*-value for each feature. We observed that the distribution of each of the 27 features was significantly different (*P *< .01) for terminator sequences and non-terminator sequences, though some features evinced a much stronger relationship than others (Fig. 2, available as supplementary data at *Bioinformatics* online). As a result, each feature, though by no means independent from the others, has some ability to differentiate terminators from non-terminators and it is reasonable to train a machine learning model to determine how best to combine the features with the goal of identifying terminators.

### 3.3 Model

Using the rich training dataset, we tuned a neural network to distinguish terminator sequences from non-terminator sequences. We then evaluated the performance of the neural network model, which we call TerminatorNet, on the testing data. All subsequent results correspond to performance on the testing data and not the training data. For terminators determined from six different Term-seq studies in *B. subtilis* or *E. coli*, we calculated TerminatorNet’s sensitivity and specificity (Fig. 2A). Across the *B. subtilis* datasets, TerminatorNet achieves an average sensitivity and specificity of 0.99 and 0.98, respectively. Across the *E. coli* datasets, TerminatorNet achieves an average sensitivity and specificity of 0.96 and 0.95, respectively. These results suggest that TerminatorNet is highly accurate in identifying terminators, correctly identifying 99% and 96% of experimentally validated terminators in *B. subtilis* and *E. coli*, respectively, with small false positive rates of 2% and 5%.

### 3.4 Comparison to other methods

We compared TerminatorNet’s performance to six leading computational tools for identifying intrinsic terminators: iTerm-PseKNC (Feng *et al.* 2019), BacTermFinder (Ghahfarokhi *et al.* 2025), termNN (Brandenburg *et al.* 2022), ARNold (Naville *et al.* 2011), RNIE (Gardner *et al.* 2011), and TransTermHP (Kingsford *et al.* 2007). Figure 2B illustrates receiver operating characteristic (ROC) curves and AUC (area under the curve) scores for different tools and Fig. 2C illustrates F1-scores for different tools all on the same set of testing data. It is worth noting that two of the tools, namely iTerm-PseKNC and ARNold, only provide binary identifications of whether a sequence contains a terminator or not rather than continuously valued scores and so ROC curves (Fig. 2B) could not be generated for these tools. Overall, TerminatorNet has the best performance in terms of ROC curve, AUC, and F1 score. Identifications from each tool for every sequence are provided with the training data (Table 2, available as supplementary data at *Bioinformatics* online) and the testing data (Table 3, available as supplementary data at *Bioinformatics* online). We also considered other performance measures for each tool, including sensitivity, specificity, precision, and Matthews correlation coefficient (Table 5, available as supplementary data at *Bioinformatics* online). Some tools, such as RNIE, identified relatively few terminators, resulting in a low false positive rate and high specificity and precision, however this came at the cost of substantially lower sensitivity. TerminatorNet was the only tool to simultaneously achieve high sensitivity and high specificity resulting in the best overall performance.

### 3.5 Contribution of different features toward terminator identification

**Figure 3:**
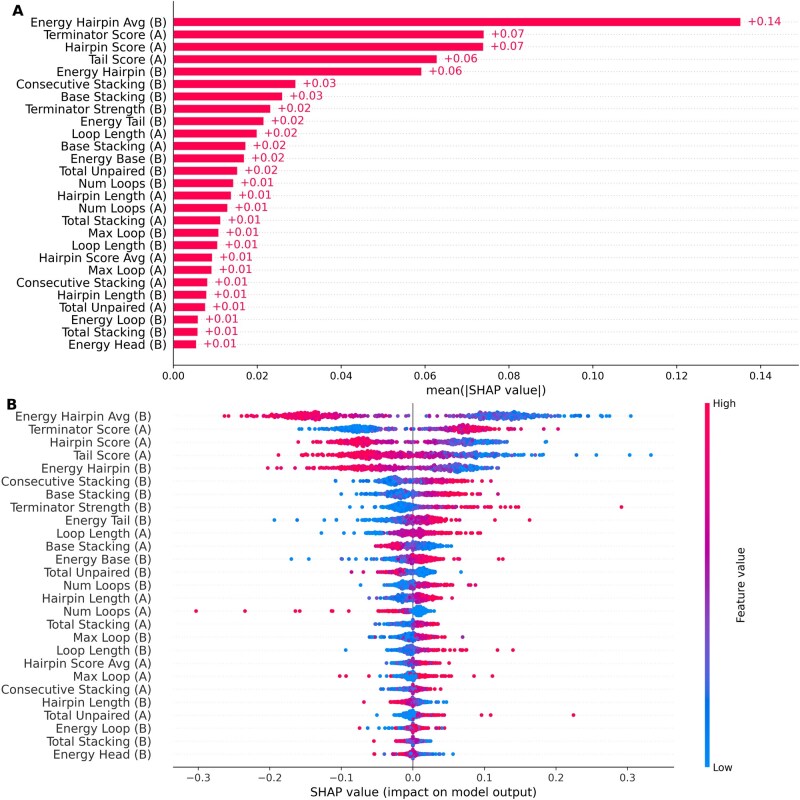
Shapley values for each of the 27 features. (A) Larger Shapley values for a feature indicate a greater contribution to the model’s output. (B) Each point in the beeswarm plot represents a sequence with the point’s color reflecting the magnitude of the feature value. Greater magnitudes along the horizontal axis reflect higher impacts on the model’s output. Negative Shapley values along the horizontal axis with larger feature values (red points on the left side of the figure) indicate that smaller values for the feature have larger model impact, e.g. for the most impactful feature Energy Hairpin Avg (B), smaller values (larger negative energies) are more suggestive of terminators. Positive Shapley values along the horizontal axis with larger feature values (red points on the right side of the figure) indicate that larger values for the feature have larger model impact, e.g. for the third most impactful feature Terminator Score (A), larger values (higher scores) are more suggestive of terminators.

For our trained neural network model, we wanted to examine the explainability of our model toward identifying terminators. Thus, we interrogated the relative contributions of different features toward the model’s output using SHAP (SHapley Additive exPlanations) analysis (Sundararajan and Najmi 2020). For each of the 27 features, we calculated Shapley values indicating the average marginal contribution of each feature. Figure 3 shows the impact of each feature on the model’s performance. As shown in Fig. 3, some features, such as Energy Hairpin Avg (B) (the free energy of the hairpin structure averaged over the number of nucleotides in the hairpin structure) make large relative contributions to the model’s output whereas other features, such as Energy Head (B) (the free energy of interaction between the head sequence and the tail sequence) make small relative contributions to the model’s output.

### 3.6 Terminators at end of genes

**Figure 4:**
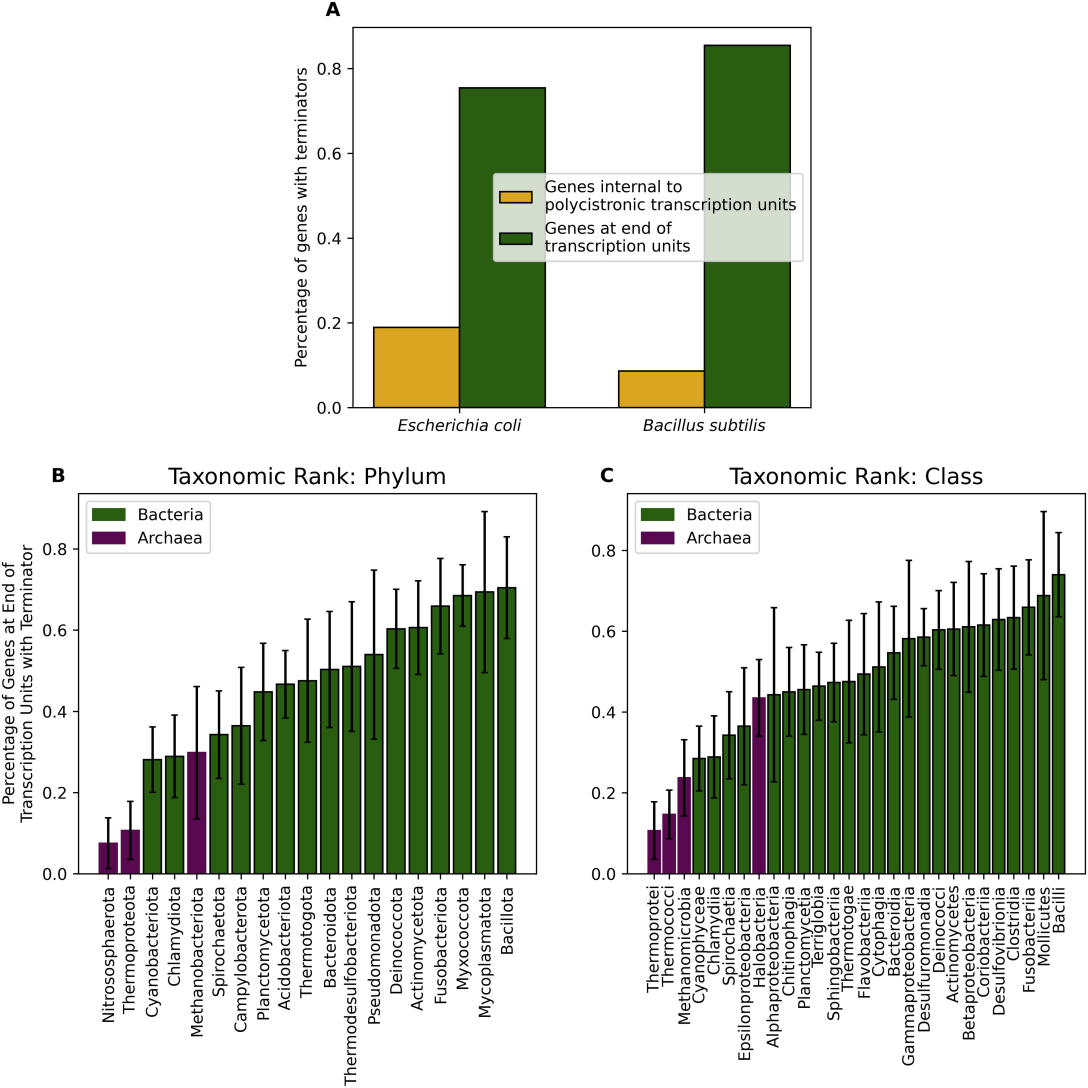
(A) For both *E. coli* and *B. subtilis*, the figure indicates the percentage of genes at the end of transcription units with identified terminators and the percentage of genes internal to polycistronic transcription units (multi-gene operons) with identified terminators. For different prokaryotic phyla (B) and classes (C), the percentage of genes at the end of transcription units with identified terminators is shown. Only phyla and classes consisting of at least 15 different species in the TerminatorNet repository are shown. Error bars reflect the standard deviation of percentages among different species within a phylum or class.

Given TerminatorNet’s accuracy in characterizing intrinsic terminators, we used it to identify terminators throughout various genomes, starting with *E. coli* and *B. subtilis*. When making terminator identifications, TerminatorNet considers only a genomic sequence and not genomic context, e.g. whether the sequence is in the vicinity of the end of an annotated gene. Not using genomic context as a feature is intentional to ensure TerminatorNet will be effective at identifying terminators in any context, including for potentially unannotated genes such as sRNAs, small proteins, and antisense transcripts. As a result, it is constructive to consider whether the terminators identified by TerminatorNet generally reside near the end of annotated transcripts. Since polycistronic transcription units (operons) are common in bacteria, we examined how often TerminatorNet’s terminator identifications corresponded to the end of a transcription unit as opposed to within a polycistronic transcription unit. Figure 4A shows that, for *E. coli* and *B. subtilis*, TerminatorNet finds a terminator near the end (within 100 nucleotides of the stop codon) of the majority of all genes that are the final gene in a transcription unit and that TerminatorNet does not find a terminator near the end (within 100 nucleotides of the stop codon) of the majority of all genes that are internal (i.e. not the final gene) to a polycistronic transcription unit. In the cases where TerminatorNet identifies terminators near the end of genes internal to operons, 19% for *E. coli* and 9% for *B. subtilis*, it is possible that these are false positive identifications but it is also possible that this reflects the dynamic nature of transcription where the entire operon is transcribed in some conditions but only portions transcribed in other conditions, e.g. because of alternative promoters and/or terminators within the operon (Güell *et al.* 2009, Sorek and Cossart 2010). Similarly, TerminatorNet identifies intrinsic terminators at the end of most but not all transcription units, 75% for *E. coli* and 85% for *B. subtilis*. For the transcription units where no intrinsic terminator is identified, it is possible that these are false negative identifications but it is also possible that an alternative form of termination, e.g. Rho-dependent termination, is being used in these cases. Our results are consistent with previous studies that have found that *E. coli* makes more use of Rho-dependent termination than *B. subtilis* (Dar and Sorek 2018, Di Salvo *et al.* 2019, Mandell *et al.* 2022). As more examples of dynamically transcribed operons are characterized in a broad range of bacteria, it will be interesting to see to what extent conditionally dependent transcription of portions of operons is more heavily used in some bacterial families than others and if TerminatorNet’s terminator identifications can help elucidate such propensities.

### 3.7 Repository of terminators throughout bacteria

We proceeded to apply TerminatorNet to thousands of bacterial genomes, starting with those labeled as “reference genomes” or “representative genomes” in NCBI’s RefSeq (Haft *et al.* 2018), in order to identify intrinsic terminators throughout the genomes. Altogether, >40 million terminators were identified. We also searched hundreds of archaeal genomes for terminators. All identified terminators, together with the genomic context of each, e.g. the terminator’s relative distance to the end of an annotated gene, are available in the TerminatorNet repository, by far the largest such database of terminators currently available. Our massive repository empowered us to consider taxonomic trends in terminator usage. We explored how often terminators occur at the end of transcription units in different phyla (Fig. 4B) and different classes (Fig. 4C). Among phyla, we found the highest level of terminator usage in bacterial Bacillota and the lowest level of terminator usage among archaeal Nitrososphaerota.

### 3.8 DNA uptake signal sequences

**Figure 5:**
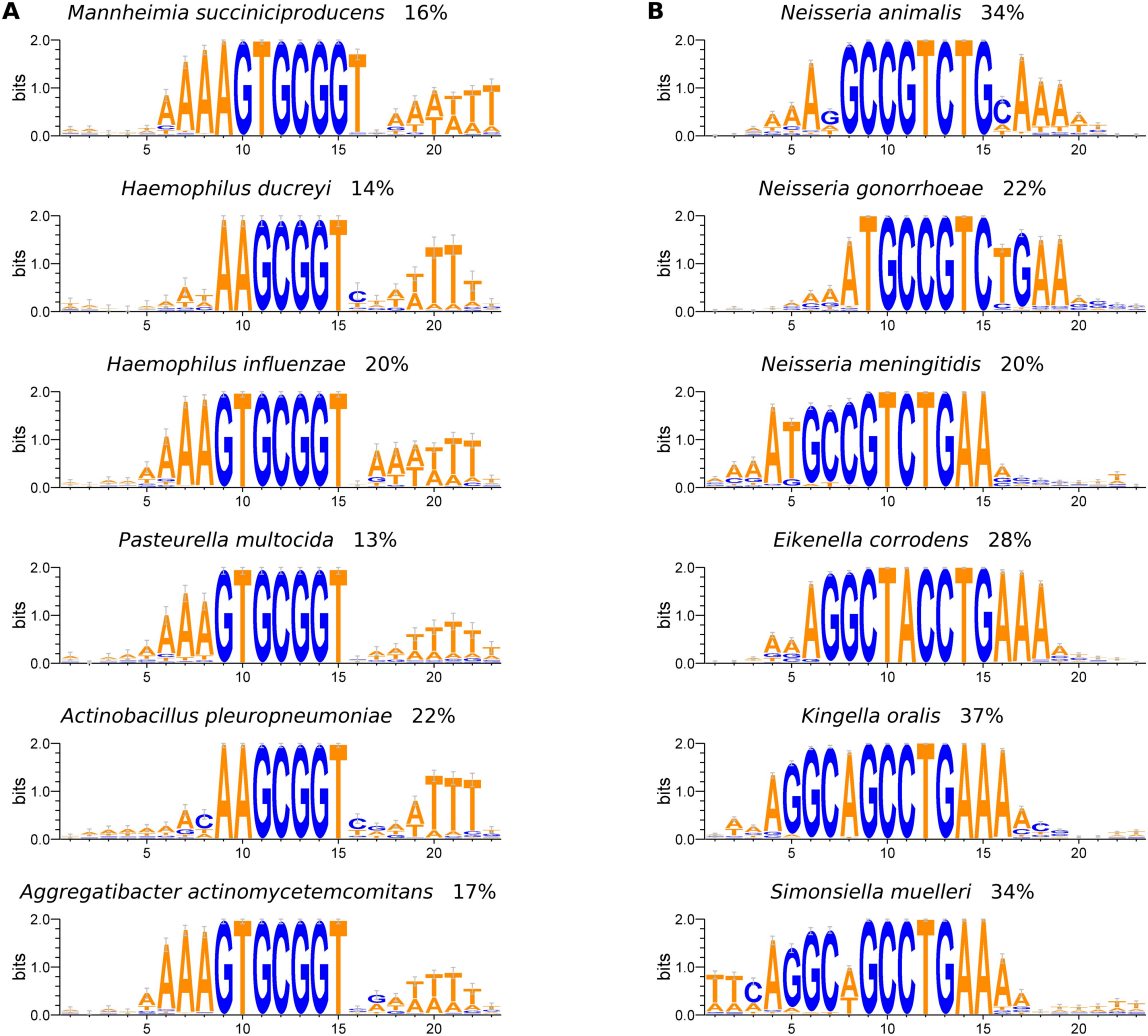
(A) Sequence motifs corresponding to uptake signal sequences (USS) occurring in identified terminators in six example species from the family Pasteurellaceae. (B) Sequence motifs corresponding to DNA uptake sequences (DUS) occurring in identified terminators in six example species from the family Neisseriaceae. Motifs were generated from all identified terminators that contain an instance of the motif. The percentage of all terminators in a genome that contain an instance of the motif is indicated.

Transformation is a mechanism for horizontal gene transfer in competent bacteria that involves the uptake of DNA from the environment (Arnold *et al.* 2022). To ensure transformation specificity, some members of *Pasteurellaceae* use an uptake signal sequence (USS, consensus AAGTGCGGT) (Mell and Redfield 2014) and some members of *Neisseriaceae* use a DNA uptake sequence (DUS, consensus GCCGTCTGAA) (Frye *et al.* 2013) that occur repeatedly throughout the genome (Dubnau and Blokesch 2019). Using the TerminatorNet repository, we searched for motifs within terminators and found DNA uptake signal sequences in terminators identified in these bacterial families (Fig. 5). Up to 22% of the terminators in some species of *Pasteurellaceae* contain instances of a DNA uptake signal sequence motif and up to 37% of the terminators in some species of *Neisseriaceae*, suggesting that these bacteria may have co-opted their frequently occurring uptake signal sequences for use in transcription termination.

## 4 Discussion

Millions of bacterial genomes have been sequenced. The ability to accurately and efficiently identify transcription terminators throughout bacterial genomes is important for many reasons. Transcription terminators and 3′ UTRs can influence transcript stability and play key roles in gene regulation. After sequencing a genome, a critical next step is identifying and annotating genes within the genome. Prokaryotic gene annotation pipelines normally use transcription initiation signals to aid in their gene annotation but not transcription termination information (Delcher *et al.* 2007, Hyatt *et al.* 2010, Lomsadze *et al.* 2018, Li *et al.* 2021). Incorporating transcription termination signals into prokaryotic gene finders may have less impact on long protein coding genes since coding sequences are compelling for discerning these genes, but the utility may be higher for more elusive genes lacking long coding regions such as sRNAs, small proteins, and antisense transcripts. The majority of all bacterial genes can be transcribed as part of polycistronic transcripts, and this co-transcription has important functional implications (Tjaden 2020). Terminator identification is a meaningful component in automated operon detection, i.e. discerning which sets of neighboring genes in a genome are co-transcribed. Currently, the extent of alternative terminators within multi-gene operons is unclear, so characterization of terminators may elucidate the prevalence of condition dependent transcription of different genes within an operon. Thus, there is value in fast and precise determination of transcription terminators in bacterial genomic sequences.

We present TerminatorNet, a system for identifying intrinsic transcription terminators, which are the primary mechanism used by bacteria to terminate their transcripts. TerminatorNet uses a neural network model trained on a large set of experimentally characterized transcription terminators from a variety of bacterial genomes. When evaluating TerminatorNet on sequences from genomes that were not used to train the model, we found that it identified 98% of terminators and had a false positive rate of 3%, which is significantly more accurate than existing approaches. Going forward, with the increasing use of high-throughput expression methodologies such as Term-seq, it will be interesting to see if the performance of computational methods for identifying terminators continues to improve with these richer sets of training data or if there is little room for improvement in computational methods given the high level of precision that we are already observing.

When considering genes that are part of operons, TerminatorNet commonly identified a terminator near the end of the last gene in the operon and uncommonly found a terminator near the end of a gene internal to the operon. Given TerminatorNet’s strong performance on a limited set of testing genomes, we proceeded to apply TerminatorNet to a broad range of genomes across the taxonomic spectrum of prokaryotes. We identified tens of millions of terminators and we observed heavy use of intrinsic transcription termination in some groups, such as Bacillota, and rare use in other groups such as archaea. We compiled this rich set of terminators into a repository. When interrogating this repository, we observed a wealth of instances of DNA uptake signal sequences, which are important components of transformation specificity for some competent bacteria, in terminators identified in *Neisseriaceae* and *Pasteurellaceae*.

While TerminatorNet is highly effective in identifying intrinsic terminators, it is important to understand some of the limitations of the approach and the data on which the model is trained and evaluated. Training data derive from high-throughput expression methodologies such as Term-seq. However, many RNA 3′ ends result not from transcription termination but from the processing of longer transcripts. Additionally, RNA stem-loops act not only as components of intrinsic terminators but also as protective barriers to RNA degradation by 3′ exonucleases (Belasco *et al.* 1985). Term-seq alone cannot differentiate between transcription terminators and endpoints of 3′-exonucleolytic processing. Further, intrinsic terminators vary widely in their termination efficiency (Cambray *et al.* 2013), and TerminatorNet is designed to capture only the existence of a functional terminator and not the terminator’s level of efficiency.

## 5 Conclusions

With the increasing use of high-throughput experiments for assaying bacterial transcription, large sets of transcription terminators have been characterized across diverse bacteria. Using these rich sources of data, we architected a neural network model to elucidate features of intrinsic transcription terminators. We find that our model is able to discern these terminators in genomic sequences with high precision. We used our model, TerminatorNet, to search thousands of prokaryotic genomes in order to compile a repository of intrinsic terminators resulting in the largest such database of transcription terminators. To facilitate use of TerminatorNet for identifying terminators in new sequences or for accessing identifications in our large repository, we have made TerminatorNet available via a web interface. We hope our system and results will be a useful resource to the myriad scientists studying transcription in bacteria.

## Supplementary Material

btag116_Supplementary_Data

## Data Availability

Training data and testing data as well as identifications from TerminatorNet and alternative tools are available as Supplementary Information. A webserver enabling execution of TerminatorNet on custom sequences as well as access to the repository of identified terminators is freely available at https://cs.wellesley.edu/~btjaden/TermNet. Code is available via GitHub at https://github.com/btjaden/TerminatorNet and Zenodo https://doi.org/10.5281/zenodo.18406126.
