# Supplementary material for: TerminatorNet: comprehensive identification of intrinsic transcription terminators in bacteria
Source: Bioinformatics. 2026 Mar 11;42(3):btag116. doi: 10.1093/bioinformatics/btag116 (PMC13020248; doi:10.1093/bioinformatics/btag116)
Supplement: btag116_Supplementary_Data [file btag116_supplementary_data.zip › Supplementary_Material.pdf]

Supplementary Material

TerminatorNet: Comprehensive Identification of Intrinsic Transcription Terminators in Bacteria

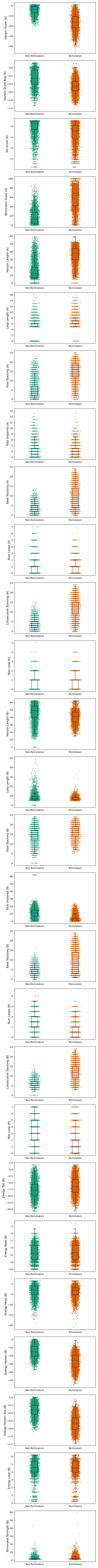
Hairpin Score (A)
Hairpin Score Avg (A)
Tail Score (A)
Terminator Score (A)
Hairpin Length (A)
Loop Length (A)
Total Stacking (A)
Total Unpaired (A)
Base Stacking (A)
Num Loops (A)
Consecutive Stacking (A)
Max Loop (A)
Hairpin Length (B)
Loop Length (B)
Total Stacking (B)
Total Unpaired (B)
Base Stacking (B)
Num Loops (B)
Consecutive Stacking (B)
Max Loop (B)
Energy Tail (B)
Energy Base (B)
Energy Head (B)
Energy Hairpin (B)
Energy Hairpin Avg (B)
Energy Loop (B)
Terminator Strength (B)
Non-Terminators
Terminators

Supplementary Figure 1

For each of the 27 features, the distribution of the feature's values is shown both for non-terminator sequences and for terminator sequences. Overlayed on each distribution is a box and whisker plot, where the horizontal line indicates the median of the distribution, the top and bottom of the box indicate the first and third quartiles of the distribution, and the whiskers extend from the box by 1.5 times the inter-quartile range.

Supplementary Figure 2

For each feature, the linear dependency between the feature and whether sequences correspond to terminators is determined using an ANOVA F-statistic and coinciding *p*-value. Larger F-statistics and smaller *p*-values indicate greater dependency.

	F-statistic	p-value
Hairpin Score (A)	12369.6	<1e-300
Hairpin Score Avg (A)	2986.9	<1e-300
Tail Score (A)	1666.4	<1e-300
Terminator Score (A)	15098.6	<1e-300
Hairpin Length (A)	10904.0	<1e-300
Loop Length (A)	1239.8	8.5E-259
Total Stacking (A)	13311.3	<1e-300
Total Unpaired (A)	35.9	2.1E-09
Base Stacking (A)	5337.3	<1e-300
Num Loops (A)	257.0	3.0E-57
Consecutive Stacking (A)	15912.8	<1e-300
Max Loop (A)	863.5	1.8E-183
Hairpin Length (B)	1084.4	6.0E-228
Loop Length (B)	460.7	2.4E-100
Total Stacking (B)	453.3	8.3E-99
Total Unpaired (B)	6442.1	<1e-300
Base Stacking (B)	3672.1	<1e-300
Num Loops (B)	5416.0	<1e-300
Consecutive Stacking (B)	10739.4	<1e-300
Max Loop (B)	5273.4	<1e-300
Energy Tail (B)	578.4	6.4E-125
Energy Base (B)	10.8	1.0E-03
Energy Head (B)	67.7	2.1E-16
Energy Hairpin (B)	5939.6	<1e-300
Energy Hairpin Avg (B)	11326.3	<1e-300
Energy Loop (B)	11.0	9.1E-04
Terminator Strength (B)	281.0	2.2E-62

TRAINING DATA

Organism	Phylum	Number of Terminators	Reference
Listeria monocytogenes	Bacillota	862	[1]
Enterococcus faecalis	Bacillota	796	[1]
Streptomyces griseus	Actinomycetota	737	[2]
Streptomyces avermitilis	Actinomycetota	705	[3]
Streptomyces coelicolor	Actinomycetota	629	[3]
Streptomyces venezuelae	Actinomycetota	573	[3]
Streptomyces tsukubensis	Actinomycetota	520	[3]
Streptomyces lividans	Actinomycetota	495	[3]
Streptomyces clavuligerus	Actinomycetota	435	[4]
Zymomonas mobilis	Pseudomonadota	206	[5]
Synechocystis sp. PCC 6803	Cyanobacteriota	165	[6]
**TOTAL**		**6123**	

TESTING DATA

Organism	Phylum	Number of Terminators	Reference
Bacillus subtilis (1)	Bacillota	1214	[7]
Bacillus subtilis (2)	Bacillota	1165	[8]
Bacillus subtilis (3)	Bacillota	984	[1]
Bacillus subtilis (4)	Bacillota	635	[9]
Escherichia coli (1)	Pseudomonadota	957	[10]
Escherichia coli (2)	Pseudomonadota	691	[11]
**TOTAL**		**5646**	

Supplementary Table 1

The 11,769 experimentally validated intrinsic transcription terminators from Term-seq experiments were split into two sets: training data from 11 bacterial species used to build a machine learning model and testing data from 2 bacterial species used to evaluate the performance of the machine learning model. For the two species in the testing set, the data derive from multiple different Term-seq studies, 4 for *B. subtilis* and 2 for *E. coli*.

Supplementary Table 2

A CSV (comma-separated values) file of training data. For each of 6,123 intrinsic terminator sequences and their 6,123 dinucleotide sampled negative control sequences, 27 feature values are provided as well the terminator identification scores for TerminatorNet and six other computational tools.

Supplementary Table 3

A CSV (comma-separated values) file of testing data. For each of 5,646 intrinsic terminator sequences and their 5,646 dinucleotide sampled negative control sequences, 27 feature values are provided as well the terminator identification scores for TerminatorNet and six other computational tools.

Feature	Description
Hairpin Score (A)	Energy associated with the folded hairpin structure identified by method (A) [12]
Hairpin Score Avg (A)	Energy of the hairpin, Hairpin Score (A), divided by the length of the hairpin, i.e., the average energy per nucleotide in the hairpin
Tail Score (A)	Measure of the distribution of uracils in the tail sequence [13]
Terminator Score (A)	Combination of Hairpin Score (A) and Tail Score (A) [12]
Hairpin Length (A)	Number of nucleotides in hairpin structure
Loop Length (A)	Number of nucleotides in hairpin loop
Total Stacking (A)	Number of nucleotides participating in basepair stacking
Total Unpaired (A)	Number of nucleotides in hairpin that are unpaired
Base Stacking (A)	Number of consecutive nucleotides involved in basepair stacking at the base of the hairpin
Num Loops (A)	Number of internal and bulge loops in the hairpin
Consecutive Stacking (A)	Number of nucleotides in the longest region of consecutive basepair stacking nucleotides
Max Loop (A)	Number of nucleotides in the longest internal or bulge loop
Hairpin Length (B)	Number of nucleotides in hairpin structure
Loop Length (B)	Number of nucleotides in hairpin loop
Total Stacking (B)	Number of nucleotides participating in basepair stacking
Total Unpaired (B)	Number of nucleotides in hairpin that are unpaired
Base Stacking (B)	Number of consecutive nucleotides involved in basepair stacking at the base of the hairpin
Num Loops (B)	Number of internal and bulge loops in the hairpin
Consecutive Stacking (B)	Number of nucleotides in the longest region of consecutive basepair stacking nucleotides
Max Loop (B)	Number of nucleotides in the longest internal or bulge loop
Energy Tail (B)	Free energy of binding of tail sequence [14]
Energy Base (B)	Free energy of stacking at base of hairpin [14]
Energy Head (B)	Free energy of head sequence [14]
Energy Hairpin (B)	Free energy of the folded hairpin structure identified by method (B) [15]
Energy Hairpin Avg (B)	Energy of the hairpin, Energy Hairpin (B), divided by the length of the hairpin, i.e., the average energy per nucleotide in the hairpin
Energy Loop (B)	Free energy of closure of hairpin loop [15]
Terminator Strength (B)	Terminator strength based on kinetic model [14]

Supplementary Table 4

Descriptions of the 27 features considered for each potential terminator sequence. A reference is included for those features described in other studies. Two different folding methods, (A) [12] and (B) [15], were used to determine hairpin structures since the two methods did not necessarily result in the same structures. The 27 features represent properties of the hairpin structures identified by method (A) or method (B).

	Sensitivity	Specificity	F1-Score	Precision	Matthews Correlation Coefficient	Area Under ROC curve (AUC)
iTerm-PseKNC	0.43	0.61	0.47	0.52	0.04	
BacTermFinder	0.55	0.99	0.70	0.97	0.59	0.93
termNN	0.97	0.47	0.77	0.64	0.51	0.94
ARNold	0.72	0.99	0.83	0.98	0.73	
RNIE	0.77	1.00	0.87	1.00	0.79	0.89
TransTermHP	0.82	0.99	0.90	0.99	0.82	0.95
TerminatorNet	0.98	0.97	0.97	0.97	0.95	0.99

Supplementary Table 5

Performance on testing data for six computational tools and TerminatorNet. Six performance measures are considered. Two tools (iTerm-PseKNC and ARNold) provide only binary classifications as opposed to continuous valued scores for whether sequences contain a terminator and, thus, ROC curves could not be generated. Shaded cells reflect the maximum value in a column, i.e., the tool that performed best for a given measure.

References

1. Dar, D., et al., *Term-seq reveals abundant ribo-regulation of antibiotics resistance in bacteria.* Science, 2016. **352**(6282): p. aad9822. PMC5756622
2. Hwang, S., et al., *System-Level Analysis of Transcriptional and Translational Regulatory Elements in Streptomyces griseus.* Front Bioeng Biotechnol, 2022. **10**: p. 844200. PMC8914203
3. Lee, Y., et al., *Genome-scale determination of 5 and 3 boundaries of RNA transcripts in Streptomyces genomes.* Sci Data, 2020. **7**(1): p. 436. PMC7738537
4. Hwang, S., et al., *Elucidating the Regulatory Elements for Transcription Termination and Posttranscriptional Processing in the Streptomyces clavuligerus Genome.* mSystems, 2021. **6**(3). PMC8269248
5. Vera, J.M., et al., *Genome-Scale Transcription-Translation Mapping Reveals Features of Zymomonas mobilis Transcription Units and Promoters.* mSystems, 2020. **5**(4). PMC7566282
6. Cho, S.H., et al., *Different Regulatory Modes of Synechocystis sp. PCC 6803 in Response to Photosynthesis Inhibitory Conditions.* mSystems, 2021. **6**(6): p. e0094321. PMC8651088
7. Mondal, S., et al., *NusA-dependent transcription termination prevents misregulation of global gene expression.* Nat Microbiol, 2016. **1**: p. 15007. PMC5358096
8. Mandell, Z.F., et al., *NusG is an intrinsic transcription termination factor that stimulates motility and coordinates gene expression with NusA.* Elife, 2021. **10**. PMC8060035
9. Chhabra, S., et al., *Analysis of mRNA Decay Intermediates in Bacillus subtilis 3' Exoribonuclease and RNA Helicase Mutant Strains.* mBio, 2022. **13**(2): p. e0040022. PMC9040804
10. Choe, D., et al., *Synthetic 3'-UTR valves for optimal metabolic flux control in Escherichia coli.* Nucleic Acids Res, 2022. **50**(7): p. 4171-4186. PMC9023263
11. Kosinski, J.G., et al., *Characterization of bacterial intrinsic transcription terminators identified with TERMITe-a novel method for comprehensive analysis of Term-seq data.* Nucleic Acids Res, 2025. **53**(12).
12. Kingsford, C.L., K. Ayanbule, and S.L. Salzberg, *Rapid, accurate, computational discovery of Rho-independent transcription terminators illuminates their relationship to DNA uptake.* Genome Biol, 2007. **8**(2): p. R22. PMC1852404
13. d'Aubenton Carafa, Y., E. Brody, and C. Thermes, *Prediction of rho-independent Escherichia coli transcription terminators. A statistical analysis of their RNA stem-loop structures.* J Mol Biol, 1990. **216**(4): p. 835-58.
14. Chen, Y.J., et al., *Characterization of 582 natural and synthetic terminators and quantification of their design constraints.* Nat Methods, 2013. **10**(7): p. 659-64.
15. Reuter, J.S. and D.H. Mathews, *RNAstructure: software for RNA secondary structure prediction and analysis.* BMC Bioinformatics, 2010. **11**: p. 129. PMC2984261
